# Commentary on the European Medicines Agency's extended mandate: Protecting public health in times of crisis and improving availability of medicines and medical devices

**DOI:** 10.1111/bcp.15567

**Published:** 2022-11-07

**Authors:** Inga Abed, Rosa Gonzalez‐Quevedo, Manuela Mura, Monica Dias, Silvy da Rocha Dias, Juan García Burgos

**Affiliations:** ^1^ Public and Stakeholders Engagement European Medicines Agency Amsterdam Netherlands; ^2^ Health Threats and Vaccines Strategy European Medicines Agency Amsterdam Netherlands; ^3^ Supply and Availability of Medicines and Devices, Regulatory Science and Innovation Task Force European Medicines Agency Amsterdam Netherlands; ^4^ Expert Panels & Groups, Committees and Quality Assurance European Medicines Agency Amsterdam Netherlands

## 1 THE COVID‐19 PANDEMIC AS A CATALYST FOR IMPROVING THE EU CRISIS RESPONSE

With the emergence of COVID‐19 in early 2019, the European Union (EU) was confronted with unexpected challenges on many fronts. On the one hand, there was an urgent need to quickly develop and authorize new medicines to treat and prevent COVID‐19, as well as devices to diagnose patients and manage disease spread^1^, ^2^, ^3^; on the other hand, the pandemic itself brought constraints to the supply chain of medicines and medical devices, impacting their availability and posing a threat to patient care.

It soon became clear that the existing emergency response structures upon which the EU relied as a whole, and which had been used to handle previous crises (Ebola,^4^ H1N1^5^), had to be adapted to a disease caused by a highly transmissible novel virus (SARS‐CoV‐2) and to the sustained demands of the pandemic.

As a result, EU institutions had to build new networks or strengthen existing networks to meet these new challenges, optimizing cooperation and coordination among different public health actors.^6^

In November 2020, the experience during the initial response to the COVID‐19 crisis and the feedback from stakeholders prompted the European Commission (EC) to propose a new framework of activities for EMA^7^ and its sister agency, the European Centre for Disease Prevention and Control (ECDC).^1^ The aim of these activities was to better address future cross‐border health threats and to better protect EU citizens in the event of future crises.

The extended mandate of EMA, which became applicable on 1 March 2022, is one of the pillars introduced by the EC to support the future European Health Union,^8^ in which all EU countries prepare and respond together to health crises, and which brings together EMA, ECDC and the newly created directorate‐general of the European Commission, the European Health Emergency Preparedness and Response Authority (HERA).^9^ This new setup will help to effectively respond to public health threats in the EU by pulling together resources and by establishing better channels of coordination and communication. Each of these 3 agencies is equipped with new mandates in areas relevant to crisis preparedness.

The EMA's extended mandate involves 3 different but complementary areas, which bring new responsibilities to EMA:

→ A coordinated EU‐level response to public health emergencies through the reinforcement and widening of the role and activities of the already existing EMA Pandemic Task Force. This strengthens the ability of the European Medicines Regulatory Network (hereby referred to as Network, composed of national medicines regulators, the EC and EMA) to face crises.
→ A stronger role of EMA in the monitoring of shortages of critical medicines, medical devices and in vitro diagnostics, both in anticipation of and during a crisis. This will improve access to medicines during and outside of a crisis.
→ A more coordinated EU expert advice on high‐risk medical devices (class IIa and III or class D^2^) and in vitro diagnostic medical devices to improve availability of devices.

## 2 EMA'S EMERGENCY TASK FORCE AS THE CORNERSTONE OF EMERGENCY RESPONSE

The Agency relies on its experts and scientific committees for the evaluation and supervision of medicines. However, a large‐scale crisis requires a dedicated structure that pools the necessary scientific expertise across the Network that can operate rapidly and flexibly, without draining the Agency's resources critical for maintaining the routine activities of medicine regulation.

During the COVID‐19 pandemic, EMA's Pandemic Task Force (renamed as the Emergency Task Force [ETF] in the new regulation) facilitated access to medicines by providing a forum for discussing marketing authorization applications and rapid scientific advices. The ETF also systematically screened evidence on medicines in the pipeline to prepare for potential marketing authorization applications. Especially relevant for EMA was the use of the rolling review to accelerate evaluation and approval of medicines.^6^ The ETF provided systematic advice during these reviews to ensure robustness and consistency of outcomes. This way, the ETF carried out important preparatory work to speed up the subsequent assessment of promising medicines by EMA's committees.

The ETF also played an important role in supporting large multinational clinical trials^10^ and by driving discussions at international level,^11^ with the aim to avoid duplication of investigations, so often seen during the pandemic, and promote clinical research to generate more robust evidence on quality, safety and efficacy of medicinal products.^12^ EMA and the pandemic task force together with the EC have facilitated and supported the creation of multinational trial networks responsible for the establishment or acceleration of adaptive pan‐European platform trials to investigate therapeutics and vaccines. An example is EU‐RESPONSE,^13^ which allowed the European expansion of the Discovery study, a clinical trial designed to evaluate medicines in hospitalized adult patients with COVID‐19; or Vaccelerate,^14^ a clinical research network for the coordination and conduct of COVID‐19 vaccine trials. Although the focus of EU‐RESPONSE and Vaccelerate is COVID‐19, these networks could be expanded to other emergencies.

By enshrining these functions into the legislation extending EMA's mandate, the temporary structures and processes used so far during the pandemic are now formalized and streamlined within a permanent body, with resources ringfenced and clear responsibilities in place.^15^ This does not only pool the relevant scientific expertise from the EU Network but also makes processes more efficient and, importantly, extends ETF's responsibilities into an active crisis preparedness role.

An important task of the ETF will be to scrutinize real‐world data emerging from the Data Analysis and Real World Interrogation Network (DARWIN EU)^16^ as well as the Vaccines Monitoring Platform (a network set up by EMA and ECDC to coordinate and oversee EU‐funded studies) and ensure that the latest effectiveness and safety data emerging from the real world are considered for regulatory decisions on medicines addressing a public health emergency. This has been well‐illustrated during the COVID‐19 pandemic where ETF assessed real‐world data as they emerged and provided recommendations that drove policy decisions on vaccination.^17^

In this context, interactions between EMA and national bodies that make decisions on the use of medicines, such as National Immunization Technical Advisory Groups for vaccines and health technology assessment bodies for therapeutics, are critical and should be maintained and expanded.

Outside of a declared public health emergency, ETF will lead regulatory activities during outbreaks of emerging pathogens that could become serious threats to provide scientific advice on and to develop strategies and requirements for medicines with a potential to address future emergencies. In addition, ETF will maintain an overview of medicines in development for future emergencies.

Before the World Health Organization declared the current monkeypox outbreak a public health emergency of international concern, ETF discussed both possible treatments and vaccines and advised on the need for additional clinical research on the disease itself. Concretely, ETF is facilitating multinational trial investigations in Europe and beyond for the vaccine Imvanex and the antiviral tecovirimat in patients affected by monkeypox. ETF is also providing scientific reviews of study protocols for clinical trial sponsors including support to applications for clinical trial authorizations, jointly with the Clinical Trials Coordination Group.^18^ As the outbreak is now a declared emergency, the ETF is working on both COVID‐19 and monkeypox pursuant to the legal provisions of the new regulation.

Moreover, EMA and ETF members have contributed to scientific and regulatory initiatives by EU bodies such as the directorate‐general of the European Commission, the HERA and the EU member states to purchase and use available medicines. The ETF has provided scientific statements on the quality specifications of imported medicines to further support the decisions of the member states when authorizing them for emergency use.^19^ Although the new regulation also grants a mandate for preparedness, the legal text focuses on management of a declared emergency. This means that any work done by the ETF outside of a declared public health emergency does not come with ringfenced resources. There is therefore scope for a more detailed and coherent legal framework in the future to also empower EMA and ETF in times of preparedness.

Finally, the high number of medicines developed and assessed for COVID‐19 as opposed to previous emergencies, as well as the duration of the COVID‐19 pandemic, have clearly highlighted the need for the EU network to become more agile and to better use available resources in Europe. Activities related to both medicines for the emergency and all other medicines need to be equally and adequately supported, and the EU network needs to ensure that the system remains sustainable during crises. Ultimately, a better preparedness and coordination before a crisis hits will soften the impact of such crisis and lead to better protection of public health in the EU and globally.

The pandemic has also seen an enhanced demand for transparency from the public, including on data supporting the approval of medicines developed in an emergency context. Public trust is the key to ensure uptake of life‐saving vaccines. EMA's extraordinary transparency measures^20^ put in place during the pandemic are intended to facilitate public scrutiny of EMA's opinions and maintain trust in the system.

## 3 IMPROVING AVAILABILITY OF MEDICINES AND MEDICAL DEVICES

Shortages of medicines have been a problem in the EU and globally long before the pandemic. With the emergence of COVID‐19, countries across the world went into lockdown, shutting down or reducing factory outputs and transport within and across borders. This affected the manufacturing, supply and distribution of medicines, leading to constraints in the global medicines supply chain. Demand also increased for some medicines used in patients with COVID‐19. These included some anaesthetics, antibiotics and muscle relaxants^21^ and also extended to medical devices such as personal protective equipment, respirators and surgical masks, diagnostics, and ventilators.^22^

The beginning of the COVID‐19 crisis exposed a gap in the coordination between EU member states, resulting in border closures and export bans on certain goods, making headlines. While the intention was to guarantee national supplies, it resulted in shortages of medicines in some EU countries and threatened the frictionless flow of goods and solidarity principle of the single market. This highlighted the limits of the existing EU framework in early 2020 in coping with immediate and imminent health emergencies.^3^

Before the pandemic, shortages in the EU were mostly handled at a national level, but EMA's role had already increased gradually over recent years. As a result of COVID‐19, EMA initiated several activities to better monitor and coordinate actions on shortages within the network (Table 1).

**TABLE 1:** European Medicines Agency activities established at the beginning of the COVID‐19 pandemic to better monitor and coordinate actions on shortages

1	Setup of the *EU executive steering group on shortages of medicines caused by major events* to provide strategic leadership for urgent and coordinated actions to prevent and mitigate supply disruptions within the EU during the pandemic
2	Regular meetings with industry associations
3	Introduction of regulatory flexibility for pharmaceutical companies to prevent/mitigate shortages
4	Launch of industry single point of contact (i‐SPOC) system, a sub‐network of contact points from marketing authorization holders to report supply issues for critical medicines
5	In addition, an enhanced fast‐track monitoring system was established to help preventing and mitigating supply issues with medicines used in COVID‐19 patients in intensive care units (ICUs) that were in high demand. This allowed regulators to detect and monitor common issues, spot patterns in medicine supply, anticipate future supply disruptions and identify EU/European economic area (EEA)‐wide measures to address disruption issues.
6	Setup of a common framework for the member states to forecast demand data in the EU/EEA, improve the forecasting of demand of medicines used in ICUs and see how to better match the estimated demand with the available supply.
7	EU SPOC network for sharing information between the member states, EMA and the EC on critical medicine shortages.

These new groups and networks have already resolved and prevented several shortages during the pandemic. An example was the shortage of protamine, a medicine used to neutralize the anticoagulant action of heparin in the treatment of haemorrhage resulting from heparin overdose. It is also used to neutralize the effect of heparin given before surgery and during extracorporeal circulation, particularly in cardiac surgery. In 2020, a major manufacturer of protamine went into liquidation and stopped operations which resulted in a Europe‐wide shortage with potentially serious impacts on patients (including cancellations of surgeries). The Single Point of Contact network reacted and issued a communication to all protamine marketing authorization holders in the EU, informing and asking them to increase production capacity to ensure the continued supply of this vital medicine during the pandemic. In addition, bilateral meetings between EMA and alternative manufacturers led to an increase in production capacity with the consequent shortage mitigation.

However, not all shortages could be resolved this way and one of the key learnings from the COVID‐19 pandemic was the need for more coordination and support amongst the member states.^6^ In this respect, the new mandate has also formalized and strengthened the ad hoc structures set up during COVID‐19.

The mandate introduced the Executive Steering Group on Shortages and Safety of Medicinal Products (the Medicine Shortages Steering Group [MSSG]), as a new executive group to coordinate major events or public health emergencies and to issue EU‐coordinated urgent actions in relation to the supply of medicines.

Shortages can be brought to the MSSG if they are considered an actual or imminent major event. This may then lead to additional reporting requirements for the marketing authorization holder, including on the cause of the shortage, current supply and demand data which will help to better allocate existing stocks and to issue recommendations on additional mitigating measures.

The MSSG has EU‐wide membership, contrary to the previous EU Executive Steering Group on Shortages of Medicines Caused by Major Events, where only several EU member states were represented. The MSSG is formally supported by the Medicines Shortages Single Point of Contact Working Party^23^ and also has formal links with ECDC and EC. Importantly, it also includes representatives of patient and healthcare professional organizations as observers.

In the area of shortages of medical devices, EMA will now monitor the supply of critical medical devices, but only during public health emergencies. There is no specific role foreseen for EMA concerning medical device shortages outside officially declared public health emergencies or major events.

By 2 February 2023, networks for medical devices mirroring the setup for medicines will have to be set up, with the necessary adaptations to the medical device sector. This is a particularly challenging piece of work as the involvement of the Agency in the area of medical devices has been limited so far and networks are not as developed or established as for medicines. Therefore, the setup of the new networks will demand considerable resource investment and strong collaboration from national authorities and economic operators.

A major tool to facilitate the detection and management of shortages will be the European shortages monitoring platform (ESMP), a centralized electronic platform to report, monitor and manage medicine shortages during crisis situations, which is scheduled for 2 February 2025. Once set up, pharmaceutical companies and the member states will use it to report shortages and relevant supply data of critical medicines during crises situations, as well as on potential supply issues that can lead to such crises. The ESMP will lead to more coordination, more effective monitoring and more prevention. However, efficient implementation will require interoperability with existing monitoring tools used at a national level. There will be challenges ahead on the information technology feasibility aspect of building a common platform linked to already existing systems while avoiding duplication of reporting processes. The overall feasibility of this project and potential solutions to already highlighted challenges will be explored over the coming months, taking into account stakeholder needs. Stakeholder needs will be collected through existing engagement forums, as well as newly set up forums such as the industry standing group, which facilitates regular exchange of views and promotes dialogue with the industry.^24^ While the use of ESMP is currently focused on preparing for and tackling crisis situations, it could be the basis for wider continuous EU‐wide monitoring outside of crises.

Tackling shortages is a complex task. The extended mandate has given EMA the regulatory tools to meet the expectations of stakeholders to move from *reporting* shortages to *avoiding* shortages. However, challenges lie ahead, especially in the area of medical devices where no pre‐existing infrastructure is in place. Continuous stakeholder engagement is essential. In addition, with a monitoring role limited to crises situations for medical devices, results are expected to have less impact in this area.

## 4 IMPROVING EVALUATION AND PERFORMANCE OF HIGH‐RISK MEDICAL DEVICES

The pandemic has also emphasized the importance of adequate supplies of medical devices across the EU and showed the need to transfer the coordination of the expert panels involved in the clinical assessment and performance evaluation of certain high‐risk medical devices to EMA. This will help to address the demands of future crises by providing timely scientific advice and technical support (e.g., in the context of the COVID‐19 pandemic, repurposing production lines for fast production of ventilators with the associated minimal technical and safety specifications).

The approval of medical devices falls under national remit and thus each member state regulates devices for its own market, with the notified bodies responsible for carrying out the conformity assessment (assessment to show the medical devices meet legal requirements, are safe and perform as intended), and issuing the certificate of conformity (CE mark) for the medical device when needed.

There are more than 500 000 medical devices on the EU market. In 2017, following safety issues with certain hip protheses^25^ and breast implants,^26^ the EC introduced new legislation on medical devices (2017 Regulations on Medical Devices (Regulation (EU) 2017/745) and on In vitro diagnostic devices (Regulation (EU) 2017/746)). This aims at improving the safety and regulatory oversight of medical devices and in vitro diagnostics in the EU, introducing new obligations for manufacturers as well as new responsibilities for EMA, the national authorities and the notified bodies.

The type of obligation and controls in place depend on the type of device. The legislation introduced a risk‐based classification system depending on the functionality and the potential impact the device can have on the human body (Table 2). While marketing of simple devices (class I, such as stethoscopes) does not require any specific evaluation, a conformity assessment by the relevant notified body is needed for those devices considered invasive (class IIa: e.g., syringes and tracheotomy tubes) or high‐risk (class IIb and III, such as ventilators and breast implants or class D which are devices used for blood screening of blood components).^2^

**TABLE 2:** Risk classes of medical devices according to the MDR (EC) 2017/745

Class I	Class IIa	Class IIb (active device intended to administer and/or remove medicinal products)	Class III
All non‐invasive devices: stethoscopes, eye occlusion plasters, wheelchairs and corrective glasses	Syringes with needles, dental fillers, tracheostomy tubes, surgical gloves, suction pumps and clamps	Ventilators, infusion pumps and anaesthesia machines	Breast implants, surgical meshes, total or partial joint replacements, spinal disc replacement and prosthetic heart valve
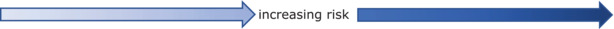			
Self‐certification	Notified body conformity assessment		
Not within scope of panels	Not within scope of panels	Within scope of panels	Within scope of panels

In addition, certain high‐risk medical devices and in vitro diagnostics belonging to class III or class IIb and certain class D diagnostics require an independent assessment by experts on the clinical evaluation performed by the notified body (Table 2).

This assessment is carried out by the EU Expert Panels, which were also introduced by the 2017 legislation and have been coordinated by the EC.

The opinions or views by the panels should be applied by the notified bodies involved in the conformity assessment of these devices before they are placed on the market. This aims at ensuring a mechanism of scrutiny on the clinical evidence for high‐risk devices. The expert panels therefore play an important role in strengthening the supervision of high‐risk medical devices and in vitro diagnostic medical devices, with the aim to ensure patient safety and help prevent future safety problems. An overview of the bodies and steps involved in the conformity assessment of high‐risk medical devices can be found in Table 3.

**TABLE 3:** Steps and bodies involved in the conformity assessment for high risk medical devices

Steps	Body	Action
**1**	Manufacturer	Submits dossier to the notified body for conformity assessment
2	Notified body	Submits clinical evaluation assessment report (CEAR) or performance evaluation report (PER) to EMA
3	Expert panels	Provides opinion or view
4	Notified body	Issues CE certificate, taking into account the expert panel evaluation

The new EMA extended mandate transfers the coordination of the expert panels from the EC to EMA. This will lead to a more integrated approach to handling public health emergencies in the EU by providing synergies with related crisis‐preparedness work for medicinal products. The Agency is well suited to manage EU expert panels due to its experience coordinating its scientific committees and scientific evaluations across Europe. The panels have been in operation since 2021 and have provided a total of 18 opinions and views on high‐risk medical devices and in vitro diagnostic medical devices (including on several diagnostic tests for SARS‐CoV‐2).^27^ The expert panels are still in the early stages of implementation and are relatively new to manufacturers, the notified bodies and the experts. Therefore, it is important to allow enough time for the expert panels and stakeholders to gather knowledge and experience with the current processes before being able to determine the overall impact of their output.

The panels will play an important role in providing greater transparency for patients and healthcare professionals on the clinical assessment done by the notified bodies. The opinions issued by the expert panels are currently published on the EC webpage,^28^ whereas, in the future, they will be published in the dedicated European database on medical devices (EUDAMED). In addition, the notified bodies will have to provide justifications if manufacturers propose to deviate from the panels' assessment, and these justifications will also have to be published.

In the longer term, the panels will also provide scientific advice to companies developing medical devices and other stakeholders. This will help the different stakeholders to consult the expert panels on the data needed for the conformity assessment, identify concerns on medical devices and help standardize the quality and performance of devices, therefore facilitating access at EU level and playing a relevant role in crisis preparedness in future public health emergencies.

Some concerns over the impartiality and scientific advice have been raised in the past.^29^, ^30^ However, EMA has always stated that as for all medicines, independence is safeguarded by a high level of transparency and stringent conflict of interest policy, which results in application of restrictions if certain interests are considered to potentially impact impartiality.^29^

The new requirements intend to increase safety while maintaining innovation and access to the EU market. This balance and the impact of the regulation, and consequently of the panels, on access to the market and innovation, will have to be measured in the coming years.

## 5 CONCLUSIONS

The pandemic has redefined the way EMA operates in times of crises. The new era in operation has shown the need for greater agility and flexibility between EMA and the European medicines regulatory network.

EMA's extended mandate brings clear benefits to the EU in terms of crisis management, which ranges from improved crises management to avoiding shortages and improving access to diagnostics and medical devices that are safe and conform to their expected function.

As part of the European Health Union, EMA's role is strengthened together with the role of ECDC, the newly created directorate‐general of the European Commission HERA and the EC. Together, they will improve the EU response to crises by increasing coordination among themselves and different public health bodies. This will ultimately offer better protection of the health of all citizens.

However, the mandate also brings new challenges for EMA, especially in an environment where workload is increased and resources are having to be concentrated on continuity of core business: preparedness needs to be improved, processes need to be carefully reviewed and redesigned to simplify administrative burden, and ensuring adequate planning and resourcing in expertise areas such as infectious diseases, medical devices and diagnostics. In the areas of medical devices, there are additional challenges due to the lack of existing networks in place.

Transparency is the key for all operations of the new mandate and so is engagement with existing stakeholders and also new stakeholder groups (such as the medical device industry). In this context, stakeholders such as healthcare professionals, patient and consumer organizations, industry and device developers have come together to exchange information on the mandate and to voice their needs and expectations and to discuss further prospects for future interactions.^31^ These discussions are continuing in existing as well as newly created fora (such as the industry standing group).

## COMPETING INTEREST

The authors report no conflicts of interest. The authors alone are responsible for the content and writing of the paper.

## CONTRIBUTORS

All authors contributed towards the writing of the text and have read and approved the final version.

## Data Availability

Data sharing is not applicable to this article because no new data were created or analysed in this study.
